# A Proving of Nux Vomica

**Published:** 1890-11

**Authors:** Ella M. Tuttle

**Affiliations:** New Berlin, N. Y.


### A PROVING OF NUX-VOM.

ELLA M. TUTTLE, M. D., NEW BERLIN, N. Y.

July 9th.—Miss M., aged thirty-four, blonde, touched the tongue to *Nux-vom.* About two hours afterward she began to have cramps in the pit of the stomach, with a feeling of nausea *in the throat.* The cramps extended to the region of the right lobe of the liver and the right side of the abdomen with a constant *fruitless* desire for stool. Fullness and heat in the pit of the stomach, followed with a feeling as if eructations would relieve but found it impossible to raise any gas. There was a dull frontal headache with a full feeling in the head, aggravated by stooping, also swimming in the brain, with a tendency to fall forward, worse by stooping. She was very irritable, desired to be let alone, and had a great aversion to any mental effort. All the symptoms worse by thinking about them.

July 10th.—Symptoms all gone except a dull feeling in the head. Bowels constipated, stool scanty.

July 11th.—A painless diarrhœa in the morning.

July 15th.—The same sick feelings returned, also a scraped sensation in the pit of the stomach. The stomach sensitive to pressure.

Since the above date the lady has had no more symptoms except alternations of constipation and diarrhœa. The most marked symptom seemed to be the nausea in the throat.

